# *PIK3CA* hotspot mutations in circulating tumor cells and paired circulating tumor DNA in breast cancer: a direct comparison study

**DOI:** 10.1002/1878-0261.12540

**Published:** 2019-09-30

**Authors:** Eleni Tzanikou, Athina Markou, Eleni Politaki, Anastasios Koutsopoulos, Amanda Psyrri, Dimitris Mavroudis, Vassilis Georgoulias, Evi Lianidou

**Affiliations:** ^1^ Analysis of Circulating Tumor Cells Lab of Analytical Chemistry Department of Chemistry University of Athens Greece; ^2^ Laboratory of Translational Oncology Medical School University of Crete Heraklion Greece; ^3^ Oncology Unit 2nd Department of Internal Medicine‐Propaedeutic Attikon University Hospital Haidari Greece; ^4^ METROPOLITAN General Hospital Athens Greece

**Keywords:** breast cancer, circulating tumor cells, circulating tumor DNA, liquid biopsy, mutation analysis, *PIK3CA*

## Abstract

Liquid biopsy analysis, mainly based on circulating tumor cells (CTCs) and circulating tumor DNA (ctDNA), provides an extremely powerful tool for the molecular profiling of cancer patients in real time. In this study, we directly compared *PIK3CA* hotspot mutations (E545K, H1047R) in EpCAM‐positive CTCs and paired plasma‐ctDNA in breast cancer (BrCa). *PIK3CA* hotspot mutations in CTCs and ctDNA were analyzed using our previously developed highly sensitive (0.05%), specific, and validated assay in plasma‐ctDNA from 77 early and 73 metastatic BrCa patients and 40 healthy donors. We further analyzed and directly compared *PIK3CA* hotspot mutations in DNAs isolated from CellSearch^®^ cartridges (CTCs) and paired plasma‐ctDNA, in 56 cases of early and 27 cases of metastatic breast cancer, and 16 corresponding primary tumors. In plasma‐ctDNA,*PIK3CA* hotspot mutations were identified in 30/77(39.0%) early and 35/73(47.9%) metastatic BrCa cases; none (0/40, 0%) of the healthy donors’ plasma‐ctDNA samples were positive. Our direct comparison study in DNAs isolated from CellSearch^®^ cartridges (CTCs) and paired plasma‐ctDNA from the same blood draws has shown a lack of concordance in early BrCa (27/56, 48.2%), while the concordance in the metastatic setting was higher (18/27, 66.6%). Our results were validated by ddPCR methodology, and the concordance between our assay and ddPCR for *PIK3CA* E545K hotspot mutation was 30/37 (81.1%). In many cases, *PIK3CA* hotspot mutations were detected in samples found to be negative for CTCs in CellSearch^®^. Our data demonstrated for the first time that (a) *PIK3CA* hotspot mutations are present at high frequencies in CTCs isolated from CellSearch^®^ cartridges and paired plasma‐ctDNA both in early and metastatic BrCa, (b) the detection and concordance of *PIK3CA* hotspot mutations between plasma‐ctDNA and CTCs are higher in the metastatic setting, (c) *PIK3CA* mutational status significantly changes after therapeutic intervention, and (d) *PIK3CA* mutation detection in CTCs and plasma‐ctDNA provides complementary information.

## Graphical abstract

Our direct comparison study on the presence of *PIK3CA* hotspot mutations (E545K, H1047R) in EpCAM‐positive CTCs and paired plasma‐ctDNA demonstrated for the first time that *PIK3CA* mutations are present at high frequencies in both CTCs and paired plasma‐ctDNA and that there is a high concordance in the metastatic setting. *PIK3CA* mutational status significantly changes after therapeutic intervention.

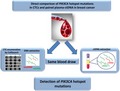

**Abbreviations**
BrCa: breast cancer
CTC: circulating tumor cells
ctDNA: circulating tumor DNA
ddPCR: droplet digital PCR
EMT: epithelial–mesenchymal transition
EpCAM: epithelial cell adhesion molecule
NGS: next‐generation sequencing
PB: peripheral blood

## 1 Introduction

Dynamic changes of tumor features over time, also known as tumor heterogeneity, are now recognized as one of the most significant issues in tumor biology (Bedard *et al*., 2014). A major limitation of the classic tissue biopsy approach is that genetically different subclones that are present at a minor frequency in the primary tumor and thus possibly not initially detected may be selected after treatment or differentially represented in the metastatic process, leading to treatment resistance (Bedard *et al*., 2014). The minimally invasive ‘liquid biopsy’ approach overcomes these limitations since by providing new ways to monitor tumor genetics and tumor dynamics in real time (Alix‐Panabières and Pantel, 2016).

Liquid biopsy has a high potential to significantly change the therapeutic strategy in cancer patients but there are still many challenging questions to be answered (Bardelli and Pantel, 2017). Detection, enumeration, and molecular characterization of circulating tumor cells (CTCs) and analysis of circulating tumor DNA (ctDNA) provide useful information regarding the individual molecular profile of each patient in real time, before and after treatment (Lianidou and Hoon, 2017). The field of liquid biopsy applications is growing exponentially, including molecular target identification, prognosis assessment, diagnosis of recurrence, monitoring of response to treatment, and monitoring of tumor genomic profiles over time (Lianidou and Hoon, 2017). Most importantly, blood‐based tests are very challenging and highly important in cases where tumor biopsies are not accessible (Ignatiadis *et al*., 2015; Lianidou *et al*., 2015; Wang *et al*., 2017). Recently, the US Food and Drug Administration (FDA) approved the first liquid biopsy‐based companion diagnostic test for the administration of first‐ to third‐generation TKI therapies in NSCLC patients, based on the presence of sensitive or resistant *EGFR* mutations in cell‐free DNA (cfDNA) (Malapelle *et al*., 2017).

Several studies so far have shown that both CTCs and ctDNA can be detected in peripheral blood of cancer patients not only in advanced but even at early stages (Bettegowda *et al*., 2014; Stathopoulou *et al*., 2002). ctDNA analysis in plasma has been suggested as an alternative to the classic biopsy approach since it essentially comprises a subtype of total cfDNA that is derived from tumor cells thus providing their genomic signature. However, there is an essential difference between CTCs and ctDNA; CTCs as viable cells circulating in peripheral blood can provide real‐time information on the metastatic spread and reveal active and possibly targetable signaling networks, while ctDNA can give specific information on the presence or absence of specific alterations deriving from the tumor, indicating therapy response or resistance (Bidard *et al*., 2018; Gold *et al*., 2015; Siravegna *et al*., 2017).

Circulating tumor cells enumeration using the CellSearch^®^ system has been approved by the FDA more than a decade ago as a prognostic marker in metastatic breast, colorectal, and prostate cancer (Riethdorf *et al*., 2018). CTC detection and enumeration are associated with progression‐free survival (PFS) and overall survival (OS) in metastatic (Bidard *et al*., 2014; Cristofanilli *et al*., 2004) and early BrCa (Lucci *et al*., 2012; Rack *et al*., 2014; Stathopoulou *et al*., 2002). Beyond enumeration, CTC molecular characterization is very important, since it can provide significant information at the gene expression, DNA methylation, and DNA mutation level (Aktas *et al*., 2016; Chimonidou *et al*., 2011, 2013; Markou *et al*., 2011, 2014; Mastoraki *et al*., 2018; Mostert *et al*., 2015; Sieuwerts *et al*., 2018; Strati *et al*., 2011). However, CTCs are highly heterogeneous, and there is a lack of a unique marker for their isolation and identification, since there are not well‐defined universal surface targets among all malignant cell types (Lianidou and Hoon, 2017). This issue is becoming more complicated when surface CTC‐enrichment targets change during epithelial‐to‐mesenchymal transition (EMT) (Gorges *et al*., 2012). Based on that, a variety of EpCAM‐independent systems have been now developed to capture and identify CTCs (Lianidou and Hoon, 2017). CTC molecular characterization can offer very important information on patient prognosis and can also identify therapeutic targets or mechanisms of resistance, such as mutations in driver genes (Bingham *et al*., 2017; Kidess‐Sigal *et al*., 2016; Liu *et al*., 2017; Pestrin *et al*., 2015; Shaw *et al*., 2017).

Plasma‐ctDNA analysis is another important component of liquid biopsy. Increased concentrations of ctDNA fragments have been detected in plasma and serum of cancer patients with various tumor types, and their presence has been correlated with unfavorable outcome (Bettegowda *et al*., 2014; Dawson *et al*., 2013; Schwarzenbach *et al*., 2011; Wan *et al*., 2017). ctDNA analysis based on tumor‐derived genetic alternations is now increasingly used for treatment purposes (Malapelle *et al*., 2017), and the large dynamic range of plasma‐ctDNA presents a considerable correlation with changes in tumor burden (Bettegowda *et al*., 2014; Dawson *et al*., 2013).

The evaluation of mutational status of specific genes in liquid biopsy material is highly informative, but requires highly sensitive and robust methodologies. Recently, our group developed and validated an ultrasensitive and highly specific methodology for the detection of *PIK3CA* hotspot mutations (E545K, H1047R) in CTCs, based on the combination of allele‐specific priming, competitive probe blocking of wild‐type amplification, asymmetric PCR, and probe melting analysis (Markou *et al*., 2014). Using this highly sensitive assay (0.05%), we have shown that *PIK3CA* hotspot mutations are present at a relatively high frequency in the EpCAM‐positive CTC fraction not only in patients with metastatic but with early BrCa as well. Our study has also shown that *PIK3CA* mutational status can change during disease recurrence or progression in BrCa patients (Markou *et al*., 2014). The aim of the present study was to use this highly sensitive methodology to directly compare the mutational status of *PIK3CA* (E545K, H1047R) in CTC‐DNAs isolated from CellSearch^®^ cartridges and paired plasma‐ctDNA from the same blood draws in patients with early and metastatic BrCa.

## 2 Materials and methods

**Figure 1:**
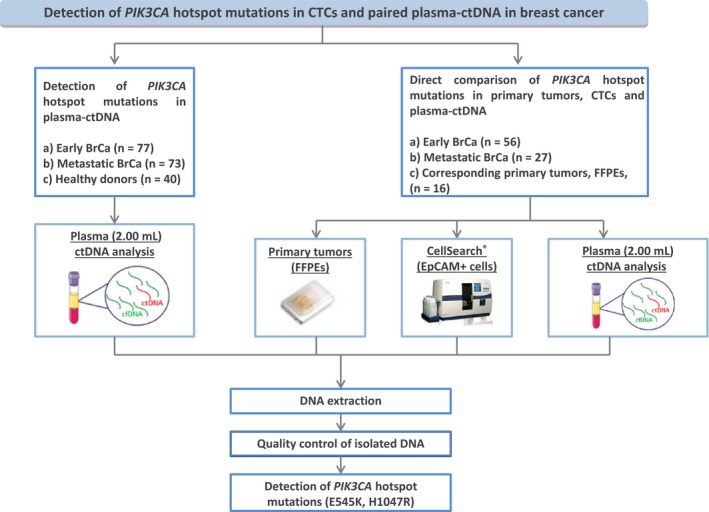
Experimental flowchart of the study.

The outline of the workflow of our study is shown in Fig. 1.

### 2.1 Clinical samples

We initially analyzed *PIK3CA* hotspot mutations in 190 plasma‐ctDNA samples from (a) patients with early BrCa (*n* = 77), (b) patients with metastatic BrCa (*n* = 73), and (c) healthy donors (all female, *n* = 40). We further performed a direct comparison study of *PIK3CA* hotspot mutations in DNAs isolated from CellSearch^®^ cartridges and paired plasma‐ctDNA in 56 samples of 43 patients with early BrCa and in 27 samples of 16 patients with metastatic breast cancer. Pretreatment and post‐treatment peripheral blood samples were available for 13 and 11 of these patients with early and metastatic BrCa, respectively, while corresponding formalin‐fixed paraffin‐embedded (FFPE) primary tumor samples were available for 16 of these patients. The study was conducted in accordance with the 1964 Declaration of Helsinki and was approved by the ethics and scientific committees of the participating Institutions. All participating patients signed an informed consent form to participate in the study, which was approved by the ethics and scientific committees of our institutions. The clinicopathological characteristics of the patients are shown in Table S1. We also included in our study 42 DNA samples isolated from CellSearch^®^ cartridges from an independent group of 17 metastatic BrCa patients, before and after first cycle of treatment. For 8 of these patients, blood sampling at progression of disease was also available. The majority of these patients were ER+/PR+/HER2−.

### 2.2 Isolation of plasma‐ctDNA

Whole blood samples were collected into venous blood collection tubes using K_2_EDTA (BD Vacutainer, Plymouth) as anticoagulant. Samples were mixed thoroughly, and plasma was isolated within 2 h from sample collection by centrifugation at 530 *g* for 10 min at room temperature. Once isolated, plasma samples were further centrifuged twice at 2000 ***g*** for 10 min, before transferring into clean 2‐mL tubes and freezing at −70 °C until further processing. The QIAamp Circulating Nucleic Acid Kit (Qiagen, Hilden, Germany) was used to isolate ctDNA from 2.00 mL of plasma according to manufacturer's instructions.

### 2.3 Isolation of gDNA from CellSearch^®^ cartridges

CellSearch^®^ analysis was performed according to the manufacturer's instructions; 7.5 mL of PB was used for each patient with metastatic disease, while in patients with early BrCa, three CellSave tubes were used for each patient (in total 22.5 mL PB). Following CTC analysis, CellSearch^®^ cartridges were stored in a dark place at 4 °C until gDNA isolation. CTCs and WBCs (prestained with antibody to CD45, pan‐CK, and DAPI) were aspirated from the CellSearch^®^ cartridge and underwent downstream gDNA extraction using the QIAamp DNA Micro Kit (Qiagen) according to manufacturer's instructions.

### 2.4 gDNA isolation from FFPEs

Formalin‐fixed paraffin‐embedded tissue sections of 10 mm containing > 80% of tumor cells were used for DNA extraction. gDNA was isolated with the QIAamp DNA FFPE Tissue Kit (Qiagen), according to the manufacturer's instructions.

### 2.5 Whole genome amplification

In the independent group of 42 clinical samples analyzed, the archival DNA material was not sufficient for downstream analysis. For this reason, a whole genome amplification (WGA) step for all these samples was included. Amplification with REPLI‐g kit (Qiagen) was performed according to the manufacturer's recommendations. A blank and DNA from human cell lines, MCF‐7 and T47D, were included for each experimental procedure as negative and positive controls, respectively. DNA concentration was determined using the Quant‐iT^™^ PicoGreen^™^ dsDNA Assay Kit (Invitrogen^™^, Waltham, MA, USA), and DNA was diluted according to the manufacturers’ manual. To verify the specificity of our assay after WGA, 10 plasma samples from healthy donors were checked before and after WGA step.

### 2.6 Sample preparation

To avoid contamination, different rooms, dedicated labware, and dedicated areas were used for all procedures. All DNA preparation and handling steps took place in specific laminar‐flow hoods under DNase‐free conditions. DNA concentration in all cases was measured in a NanoDrop 1000 spectrophotometer (Thermo Scientific, Waltham, MA, USA) , calibrated with the recommended CF‐1 (concentrated aqueous potassium dichromate, K_2_Cr_2_O_7_) standard solution. The isolated DNA samples were stored at −70 °C until further use.

### 2.7 *PIK3CA* hotspot mutation analysis

The detection of *PIK3CA* hotspot mutations (E545K, H1047R) was based on our previously developed and validated ultrasensitive assay (analytical sensitivity 0.05%) (Markou *et al*., 2014).

### 2.8 Quality control

Synthetic gene fragments were synthesized as gBlocks by Integrated DNA Technologies (Coralville, IA, USA) and were used as positive controls for *PIK3CA* hotspot mutations (sequences are available upon request). Synthetic oligos for E545K and H1047R mutation sites were designed so that each sequence was represented by a unique gBlock that mutation position would be included. Lyophilized gBlocks were suspended in Tris/EDTA buffer to a stock concentration of 10 ng·mL^−1^ and were further diluted to working concentrations as needed. Also, two negative controls were included in each experimental procedure, one as negative control of PCR reaction (blank) and the second one as wild‐type control, which contained wild‐type as DNA template. All samples were checked for their DNA integrity prior to analysis, by amplifying a region of *PIK3CA* exon 20 that includes the hotspot mutation site.

### 2.9 Spiking experiments

To further verify the analytical sensitivity of the assay, we collected peripheral blood samples from two healthy donors in 2 different types of tubes: (a) K2EDTA (BD Vacutainer) and (b) CellSave (Menarini Silicon Biosystems). In both cases, the first 5 mL of blood was discarded to avoid skin epithelial cell contamination. In each tube, MCF‐7 cells (100 cells enumerated in a Malassez Hemocytometer) were spiked into peripheral blood (10 mL in EDTA tube and 7.5 mL in CellSave tube), and mixed immediately after spiking. In the EDTA tube, CTC isolation was performed by adding red cell lysis buffer and capture beads, coated with the monoclonal antibody BerEP4 against the human epithelial antigen EpCAM. In the CellSave tube, CellSearch^®^ analysis was performed according to the manufacturer's instructions. Following CTC analysis, CellSearch^®^ cartridges were used for downstream analysis of EpCAM‐positive CTCs. In both cases, DNA was isolated using QIAamp DNA Micro Kit and DNA concentration was measured in a NanoDrop 1000 spectrophotometer that was calibrated with the recommended CF‐1 standard solution.

### 2.10 Droplet digital PCR for the detection of *PIK3CA* E545K hotspot mutation

We analyzed 37 of these DNA samples using droplet digital PCR (ddPCR) technology for *PIK3CA* E545K hotspot mutation. All clinical samples were analyzed with the QX200 Droplet Digital PCR System (Bio‐Rad Laboratories, Inc., Hercules, CA, USA) using TaqMan hydrolysis probes for detecting and quantifying wild‐type *PIK3CA*, as well as *PIK3CA* E545K in exon 9. One probe was specific for the wild‐type sequence (wild‐type reference assay/HEX, Channel 2), and the other was specific for each *PIK3CA* E545K hotspot mutation (mutation assay/FAM, Channel 1). The PCR mix for one well was set up by mixing 2 μL DNA sample, 10 μL 2 × ddPCR Supermix for Probes (No dUTP) (Bio‐Rad Laboratories), 1 μL 20 × target primers/probe (FAM), 1 μL 20 × reference primers/probe (ΗΕΧ), in a reaction volume of 20 μL, adjusted with sterile water, according to the manufacturer's instructions. The cycling profile of the *PIK3CA* mutant detection assay was as follows: 95 °C for 10 min (1 cycle), 94 °C for 30 s, and 55 °C for 60 s (55 cycles), and infinite hold at 12 °C. Each experimental procedure included a negative control (nontemplate control) and a positive control (MCF‐7 cell line), which contained enough mutant and wild‐type DNA to create a double‐positive cluster of greater than 100 droplets. PCR products were loaded onto the QX200 Droplet Reader (Bio‐Rad Laboratories, Inc.), and the data were analyzed with QuantaSoft Software.

### 2.11 Statistical analysis

Statistical analysis was performed via SPSS version 24.0 (IBM^®^ SPSS^®^ Statistics, Armonk, NY, USA). A *P*‐value ≤ 0.05 was considered to be significant. Graphics were generated with MS Excel 2010 (Microsoft Corporation, Seattle, WA, USA). Agreement between the presence of *PIK3CA* hotspot mutations in DNAs isolated from CellSearch^®^ cartridges and paired plasma‐ctDNA was assessed by using the chi‐square test and McNemar test.

## 3 Results

### 3.1 Detection of *PIK3CA* hotspot mutations in plasma‐ctDNA

**Figure 2:**
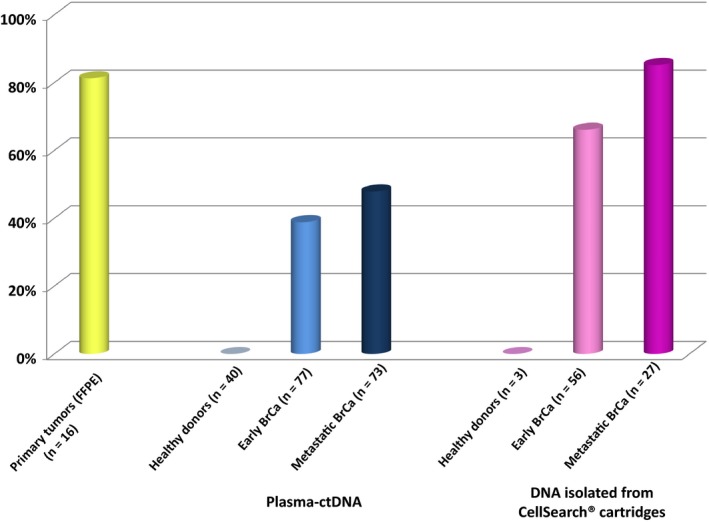
*PIK3CA* hotspot mutations in primary tumors, DNA isolated from plasma‐ctDNA and CellSearch cartridges.

We analyzed in total 190 plasma‐ctDNA samples for *PIK3CA* hotspot mutations from (a) early BrCa (*n* = 77), (b) metastatic BrCa (*n* = 73), and (c) healthy donors (all female, *n* = 40). We first evaluated the specificity of the *PIK3CA* hotspot mutations assay in plasma‐ctDNA samples from 40 female healthy donors. None of the plasma‐ctDNA (0/40, 0%) was found positive for both *PIK3CA* hotspot mutations (Fig. 2). Moreover, to verify further the specificity of this procedure, a WGA step was also included prior to analysis and 10 plasma samples from healthy donors were checked before and after WGA in order to ensure that no false‐positive results are detected. Indeed, both *PIK3CA* hotspot mutations (E545K, H1047R) were not detected before and after WGA using our highly sensitive assay. Concerning analytical sensitivity, our spiking experiment results have clearly shown that PIK3CA E545K hotspot mutation was detected by using our laboratory‐developed assay. *PIK3CA* hotspot mutations were identified in plasma‐ctDNA in 30/77(39.0%) and in 35/73(47.9%) cases of early BrCa and metastatic BrCa, respectively. In early BrCa, *PIK3CA* E545K hotspot mutation was identified in 30/77(39.0%) plasma‐ctDNA samples, and *PIK3CA* H1047R hotspot mutation was identified in 7/77(9.1%). In metastatic BrCa, 30/73(41.1%) ctDNA samples were found positive for *PIK3CA* E545K hotspot mutation and 15/73(20.5%) were found positive for *PIK3CA* H1047R hotspot mutation.

### 3.2 Direct comparison of *PIK3CA* hotspot mutations in DNAs isolated from CellSearch^®^ cartridges and paired plasma‐ctDNA

**Figure 3:**
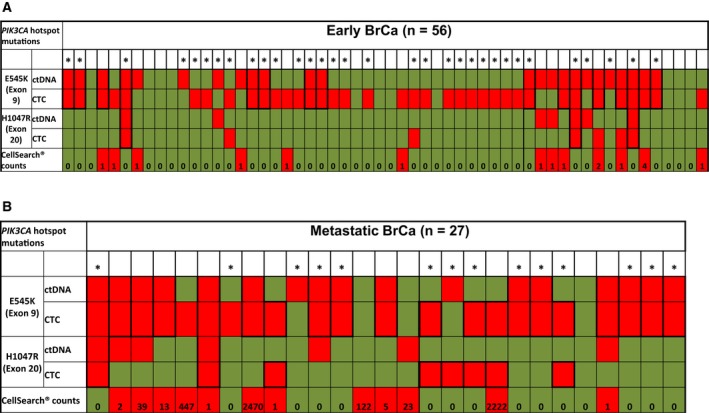
Direct comparison study of *PIK3CA* hotspot mutations in DNA isolated from CellSearch^®^ cartridges and paired plasma‐ctDNA from (A) early and (B) metastatic BrCa patients.

Circulating tumor cells enumeration using the CellSearch^®^ and *PIK3CA* mutational analysis in plasma and DNAs isolated from CellSearch^®^ cartridges were performed in a subgroup of these patients, using the same blood draw (same date and time), more specifically: (a) in 56 samples from 43 patients with early BrCa, (b) in 27 samples from 16 patients with metastatic BrCa, and (c) in 3 PB samples from healthy blood donors. None of DNA samples isolated, in exactly the same way, from CellSearch^®^ cartridges from peripheral blood of these three healthy donors was found positive for *PIK3CA* hotspot mutations. In early BrCa, *PIK3CA* hotspot mutations were detected in 37/56(66.1%) DNA samples isolated from CellSearch^®^ cartridges (Fig. 2). More specifically, *PIK3CA* E545K hotspot mutation was detected in 37/56(66.1%) DNA samples isolated from CellSearch^®^ cartridges, whereas *PIK3CA* H1047R hotspot mutation was detected in 7/56(12.5%) (Fig. 3). CellSearch^®^ detected at least one CTC in 13/56(23.2%) samples. *PIK3CA* hotspot mutations were identified in 9/13(69.2%) DNAs samples isolated from CellSearch^®^ cartridges where at least one CTC was detected. Moreover, in 28/43(65.1%) cases, *PIK3CA* hotspot mutations were detected in CellSearch^®^ cartridges that were found negative for CTCs (Fig. 3). In metastatic BrCa, *PIK3CA* hotspot mutations were detected in 23/27(85.2%) DNA samples isolated from CellSearch^®^ cartridges (Fig. 2). *PIK3CA* E545K hotspot mutation was detected in 22/27(81.5%) DNAs isolated from CellSearch^®^ cartridges, whereas *PIK3CA* H1047R hotspot mutation was detected in 8/27(29.6%) (Fig. 3). CellSearch^®^ detected at least one CTC in 12/27(44.4%) samples, and *PIK3CA* hotspot mutations were identified in 10/12(83.3%) DNAs isolated from CellSearch^®^ cartridges positive for CTCs. *PIK3CA* hotspot mutations were also detected in 13/15(86.7%) cases that were found negative for CTCs by CellSearch^®^. In two cases, where CellSearch^®^ detected 122 and 23 CTCs, respectively, *PIK3CA* hotspot mutations were not detected (Fig. 3).

Paired plasma‐ctDNA samples were analyzed for the same *PIK3CA* hotspot mutations. In early BrCa, *PIK3CA* hotspot mutations were detected in 24/56(42.9%) paired plasma‐ctDNA samples. More specifically, *PIK3CA* E545K hotspot mutation was detected in 24/56(42.9%), whereas *PIK3CA* H1047R hotspot mutation was detected in 7/56(12.5%) plasma‐ctDNA samples (Fig. 3). In metastatic BrCa, *PIK3CA* hotspot mutations were detected in 18/27(66.7%) paired plasma‐ctDNA samples. *PIK3CA* E545K hotspot mutation was detected in 17/27(63.0%) plasma‐ctDNA samples, whereas *PIK3CA* H1047R hotspot mutation was detected in 7/27(25.9%) (Fig. 3). Both *PIK3CA* hotspot mutations were identified in the same plasma samples in seven early and six metastatic BrCa cases.

In early BrCa, *PIK3CA* hotspot mutations were detected in 37/56(66.1%) DNA samples isolated from CellSearch^®^ cartridges and in 24/56(42.8%) paired plasma‐ctDNA samples. In total, in these 56 samples, the concordance of *PIK3CA* hotspot mutations between DNAs isolated from CellSearch^®^ cartridges and paired plasma‐ctDNA samples was 27/56 (48.2%) (Table 1). In metastatic BrCa, *PIK3CA* hotspot mutations were detected in 23/27(85.2%) DNA samples isolated from CellSearch^®^ cartridges and in 18/27(66.7%) paired plasma‐ctDNA samples. In total, in these 27 samples, the concordance of *PIK3CA* hotspot mutations between DNA isolated from CellSearch^®^ cartridges and paired plasma‐ctDNA samples was 18/27 (66.6%) (Table 1). The correlation of *PIK3CA* hotspot mutations and clinicopathological characteristics of early and metastatic BrCa patients used in the direct comparison study is shown in Table S2.

**Table 1:** Direct comparison of *PIK3CA* hotspot mutations in ctDNA and CellSearch^®^ cartridges isolated from early (*n* = 56) and metastatic (*n* = 27) BrCa patients

*PIK3CA* hotspot mutations (early breast cancer)				
*N* = 56	CellSearch^®^ cartridges			
ctDNA	Neg	Neg	Pos	Total
		11	21	32
	Pos	8	16	24
	Total	19	37	56
Concordance: (27/56) 48.2% (*P* = 0.582, chi‐square test)				

*PIK3CA* hotspot mutations (metastatic breast cancer)				
*N* = 27	CellSearch^®^ cartridges			
ctDNA	Neg	Neg	Pos	Total
		2	7	9
	Pos	2	16	18
	Total	4	23	27
Concordance: (18/27) 66.6% (*P* = 0.407, chi‐square test)				

### 3.3 *PIK3CA* mutational status in CTCs and paired plasma‐ctDNA changes before and after treatment

#### 3.3.1 Early BrCa

**Figure 4:**
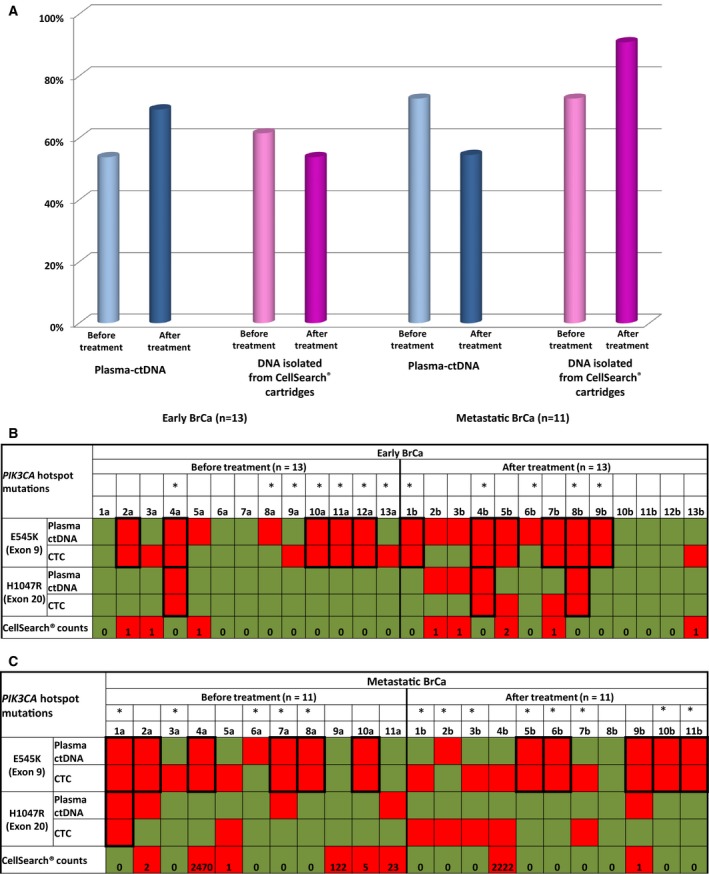
(A) *PIK3CA* hotspot mutations in DNA isolated from CellSearch^®^ cartridges and paired plasma‐ctDNA, before and after treatment from (i) early (*n* = 13) and (ii) metastatic (*n* = 11) BrCa patients. Heatmap for (B) early and (C) metastatic BrCa patients.

In early BrCa, *PIK3CA* mutational status was evaluated in 13 cases where peripheral blood samples were available before and after treatment. Before treatment, *PIK3CA* hotspot mutations were detected in plasma‐ctDNA in 7/13(53.8%), and in corresponding DNAs isolated from CellSearch^®^ cartridges in 8/13(61.5%) cases. After treatment, *PIK3CA* hotspot mutations were detected in plasma‐ctDNA in 9/13(69.2%), and in DNAs isolated from CellSearch^®^ cartridges in 7/13(53.8%) cases (Fig. 4A). Before treatment, *PIK3CA* E545K hotspot mutation was detected in 8/13(61.5%) DNA samples isolated from CellSearch^®^ cartridges and in 7/13(53.8%) paired plasma‐ctDNA samples. However, only one sample was positive for *PIK3CA* H1047R hotspot mutation in both DNA isolated from CellSearch^®^ cartridge and the paired plasma‐ctDNA samples 1/13(7.7%) (Fig. 4B). After treatment, *PIK3CA* E545K hotspot mutation was detected in 7/13(53.8%) DNAs isolated from CellSearch^®^ cartridges and in 9/13(69.2%) paired plasma‐ctDNA samples (Fig. 4B). *PIK3CA* H1047R hotspot mutation was detected in 4/13(30.8%) DNA isolated from CellSearch^®^ cartridges and in 4/13(30.8%) paired plasma‐ctDNA samples. After treatment, five patients (#2, #4, #5, #8, and #13) retained their initial *PIK3CA* hotspot mutation status in plasma‐ctDNA and four patients (#4, #6, #9, and #13) in DNA isolated from CellSearch^®^ cartridges (Fig. 4B). In five cases (#1, #3, #6, #7, and #9), *PIK3CA* hotspot mutations were detected only in post‐treatment plasma‐ctDNA samples, but not before whereas in three cases (#10, #11, and #12) *PIK3CA* hotspot mutations were detected in plasma‐ctDNA only before treatment. In four cases (#1, #5, #7, and #8), *PIK3CA* hotspot mutations were detected only in post‐treatment DNA samples isolated from CellSearch^®^ cartridges, whereas their corresponding pretreatment samples were found negative. However, there were five cases (#2, #3, #10, #11, and #12) where *PIK3CA* hotspot mutations were detected in pretreatment but not in the corresponding post‐treatment samples. According to our results, after treatment, more plasma‐ctDNA samples but less DNA samples isolated from CellSearch^®^ cartridges were found positive for *PIK3CA* hotspot mutations.

#### 3.3.2 Metastatic BrCa

In metastatic BrCa, *PIK3CA* mutational status was evaluated in 11 cases where peripheral blood samples were available before and after completion of first‐line treatment. Before treatment, *PIK3CA* hotspot mutations were detected in plasma‐ctDNA in 8/11(72.7%), and in corresponding DNAs isolated from CellSearch^®^ cartridges in 8/11(72.7%) cases (Fig. 4A). *PIK3CA* E545K hotspot mutation was detected in 8/11(72.2%) DNA isolated from CellSearch^®^ cartridges and in 7/11(63.6%) paired plasma‐ctDNA samples (Fig. 4C). *PIK3CA* H1047R hotspot mutation was detected in 2/11(18.2%) DNA isolated from CellSearch^®^ cartridges and in 4/11(36.6%) paired plasma‐ctDNA samples (Fig. 4C). After first‐line treatment, *PIK3CA* E545K hotspot mutation was detected in 9/11(81.8%) DNA isolated from CellSearch^®^ cartridges and in 6/11(54.5%) paired plasma‐ctDNA samples. *PIK3CA* H1047R hotspot mutation was detected in 5/11(45.5%) DNA isolated from CellSearch^®^ cartridges and in 1/11(9.1%) paired plasma‐ctDNA samples. After first‐line treatment, in five patients (#1, #2, #3, #6, and #10), the same *PIK3CA* hotspot mutations were detected in plasma‐ctDNA and in seven patients (#1, #2, #3, #4, #5, #6, and #10) the same *PIK3CA* hotspot mutations were detected in DNA isolated from CellSearch^®^ cartridges (Fig. 4C). For two patients (#5 and #9), *PIK3CA* hotspot mutations were detected only in post‐treatment plasma‐ctDNA, but not before treatment. On the contrary, in four cases (#1, #4, #7, and #8), *PIK3CA* hotspot mutations were detected only in pretreatment plasma‐ctDNA samples, but not after treatment. In three cases (#6, #9, and #11), *PIK3CA* hotspot mutations were detected only in DNA isolated from CellSearch^®^ cartridges post‐treatment, whereas the corresponding pretreatment samples were found negative. There was only one case (#8) where *PIK3CA* mutation was detected only before pretreatment sample, but not after treatment. According to our results, after treatment, more DNA samples isolated from CellSearch^®^ cartridges but less plasma‐ctDNA samples were found positive for *PIK3CA* hotspot mutations.

### 3.4 Comparison of *PIK3CA* hotspot mutations in primary tumors, corresponding DNA isolated from CellSearch^®^ cartridges and paired plasma‐ctDNA

For 16 cases, DNA isolated from corresponding primary tumors (FFPEs) was also available for comparison studies (Table 2). *PIK3CA* hotspot mutations were identified in 13/16(81.3%) of these FFPE samples (Fig. 2); *PIK3CA* E545K hotspot mutation was detected in 9/16(56.3%), and *PIK3CA* H1047R hotspot mutation was detected in 11/16(68.8%) (Table 2). In all cases where *PIK3CA* E545K hotspot mutation was detected in FFPEs, it was also detected in DNA isolated from CellSearch^®^ cartridges. Moreover, there were six cases (#4, #6, #9, #10, #14, and #16) where *PIK3CA* E545K mutation was detected *de novo* in DNA isolated from CellSearch^®^ cartridges, but was not detected in FFPEs. In three of these cases (#4, #14, and #16), E545K was also detected in paired plasma‐ctDNA samples. A high concordance for *PIK3CA* hotspot mutations between FFPE and DNAs isolated from CellSearch^®^ cartridges was found (12/16, 75.0%), whereas this concordance was much lower between FFPE and plasma‐ctDNA samples (6/16, 37.5%) (Table 2).

**Table 2:** *PIK3CA* hotspot mutations in primary tumors, DNA isolated from CellSearch^®^ (CS) cartridges and plasma‐ctDNA from BrCa patients (*n* = 16)

*PIK3CA* hotspot mutations								
# ID	E545K			H1047R			CellSearch^®^ (CS) counts	Status
	Primary tumor	ctDNA	CTCs (CS)	Primary tumor	ctDNA	CTCs (CS)		
1	+	−	+	+	−	−	0	Early
2	−	−	−	+	−	−	0	Early
3	+	−	+	+	−	−	0	Early
4	−	+	+	+	−	−	0	Early
5	+	−	+	+	−	−	1	Early
6	−	−	+	+	−	−	0	Early
7	+	+	+	+	+	−	0	Metastatic
8	+	+	+	+	−	−	NA	Early
9	−	−	+	−	−	+	0	Early
10	−	−	+	+	−	−	0	Early
11	+	+	+	+	+	−	2	Metastatic
12	+	−	+	+	−	−	0	Metastatic
13	+	−	+	−	−	−	447	Metastatic
14	−	+	+	−	+	+	1	Metastatic
15	+	+	+	−	+	−	39	Metastatic
16	−	+	+	−	+	+	0	Metastatic

### 3.5 Comparison study between the combination of allele‐specific, asymmetric rapid PCR and melting analysis assay and ddPCR for the detection of *PIK3CA* E545K hotspot mutation

The results of the clinical sample analysis were based on the definition of threshold line for each subpopulation of mutant or wild‐type droplets. All positive droplets, those above the threshold line of 3.500 droplets, are scored as positive, whereas the negative droplets those above the threshold line of 3.500 are scored as negative (background). In case of clinical samples, at least one droplet of *PIK3CA* E545K DNA‐mutated sequence was considered as positive sample for *PIK3CA* E545K hotspot mutation by ddPCR.

For this study, we analyzed in total by ddPCR 37 samples: 17 genomic DNAs isolated from CellSearch^®^ cartridges (EpCAM‐positive cells) and 20 plasma‐ctDNA samples for *PIK3CA* E545K mutation. More specifically, 14 and 3 DNAs isolated from CellSearch^®^ cartridges (EpCAM‐positive cells) from early and metastatic patients, and 11 and 9 plasma‐ctDNA samples from early and metastatic patients, respectively, were used for this study.

In total, the concordance between our assay and ddPCR for *PIK3CA* E545K hotspot mutation was 30/37 (81.1%) (Table 3). More specifically, 21 samples were found positive by both methods, and 9 samples found negative by both methods. There were only 2 samples that were found positive by our method and negative by ddPCR, and 5 samples that were found positive by ddPCR and negative by our method. Our data support that our assay and ddPCR give comparable results (*P* = 0.001).

**Table 3:** Comparison study between the combination of allele‐specific, asymmetric rapid PCR and melting analysis assay and droplet digital PCR for detecting *PIK3CA* E545K hotspot mutation in early and metastatic BrCa patients

Combination of allele‐specific, asymmetric rapid PCR and melting analysis assay (Markou *et al*., 2014)	ddPCR		
Neg	Neg	Pos	Total
	9	5	14
Pos	2	21	23
Total	11	26	37
Concordance: (30/37) 81.8% (*P* = 0.001, chi‐square test)			

### 3.6 *PIK3CA* hotspot mutations in CTCs from an independent group of DNA samples isolated from CellSearch^®^ cartridges

In the independent patient group studied, *PIK3CA* hotspot mutations were detected in 25/42(59.5%) DNA samples isolated from CellSearch^®^ cartridges most of which were positive for CTCs (Fig. S1). *PIK3CA* E545K hotspot mutation was detected in 12/42(28.6%) DNAs isolated from CellSearch^®^ cartridges, whereas *PIK3CA* H1047R hotspot mutation was detected in 17/42(40.5%). CellSearch^®^ detected at least one CTC in 37/42(88.1%) samples, and *PIK3CA* hotspot mutations were identified in 22/37(59.5%) DNAs isolated from CellSearch^®^ cartridges positive for CTCs. Moreover, *PIK3CA* hotspot mutations were also detected in 3/5(60%) cases that were found negative for CTCs by CellSearch^®^. These data are in concordance with our previous results, indicating that *PIK3CA* mutations are also present in EpCAM‐positive cells where CellSearch^®^ gave negative results for the presence of CTCs. In this independent patient group, we found in total 4/42(9.5%) samples where both *PIK3CA* mutations were detected. As can be seen in Fig. S1, in all these four cases, CTCs were detected in the CellSearch^®^. Moreover, in 15/42(35.7%) cases where CellSearch^®^ was positive for CTCs, no *PIK3CA* mutations were detected.

## 4 Discussion

Liquid biopsy is now becoming a highly important tool for following up cancer patients in real time with a variety of solid tumors (Alix‐Panabières and Pantel, 2016; Bardelli and Pantel, 2017; Bedard *et al*., 2014; Bettegowda *et al*., 2014; Ignatiadis *et al*., 2015; Lianidou and Hoon, 2017; Lianidou *et al*., 2015; Malapelle *et al*., 2017; Stathopoulou *et al*., 2002; Wang *et al*., 2017). However, some questions still need to be answered (Bardelli and Pantel, 2017). One common question is whether CTCs and ctDNA give identical or complementary information, and which of the two is the best choice to follow up patients over time. *PIK3CA* mutational status has already been studied in CTCs and plasma‐ctDNA using different methodologies such as NGS and ddPCR; however, there are no studies till now where *PIK3CA* mutational status was evaluated in CTC and plasma‐ctDNA in identical blood draws using the same methodology. In the present study, we addressed this question for the first time by performing a direct comparison study of *PIK3CA* mutational status (E545K, H1047R) in DNA isolated from CellSearch^®^ cartridges and paired plasma‐ctDNA from the same blood draws in early and metastatic BrCa, using our previously described highly sensitive real‐time PCR methodology (Markou *et al*., 2014).

PI3K is one among the most important downstream molecules in the pathway of tyrosine kinase growth factor receptors and one of the most promising targets for personalized medicine. *PIK3CA* mutations have been reported in 18%–40% (Barbareschi *et al*., 2007) of BrCa cases; three ‘hotspot’ mutations (E542K, E545K, and H1047R) comprise more than 80% (Campbell *et al*., 2004) of all *PIK3CA* mutations and are localized in exons 9 and 20. Moreover, early clinical studies have indicated that the presence of *PIK3CA* mutations is linked to the acquirement of enhanced sensitivity to PI3K pathway inhibitors (Janku *et al*., 2011). Although the presence of *PIK3CA* mutations in archival tissues has been evaluated in many clinical trials such as BOLERO‐2, FERGI, BELLE‐2, and BELLE‐3, the PFS benefit from PI3K or mTOR inhibitors seems to be largely maintained irrespectively of *PIK3CA* genotype (Hortobagyi *et al*., 2016); it is important to note that the detection limit of the next‐generation sequencing methodology used in this study for mutation allele frequency (MAF) was 5%.

However, after taking into consideration the advantages of noninvasive liquid biopsy analysis, we expect that identifying key mutations in CTCs and plasma‐ctDNA from peripheral blood samples may provide more actionable information on the molecular profile of metastatic tumors. Results of the BELLE‐2 and BELLE‐3 clinical studies, based on the combination of a pan‐PI3K inhibitor (buparlisib) with endocrine therapy, indicated a better PFS in the *PIK3CA* mutant subgroup identified through ctDNA analysis, but not for the *PIK3CA* mutant subgroup as identified in primary tumors (Baselga *et al*., 2017; Di Leo *et al*., 2018). However, the BOLERO‐2 study which examined the benefit of an inhibitor of the PI3K/AKT/mTOR pathway (everolimus), has shown a benefit for patients when everolimus was added to endocrine therapy regardless of the presence of a *PIK3CA* mutation (H1047R, E545K, and E542K) in either tumor tissues or ctDNA samples analyzed by droplet digital PCR (ddPCR), suggesting that *PIK3CA* mutation status is not a predictive determinant for everolimus benefit (Moynahan *et al*., 2017). mTOR, however, is downstream of PI3K, and studies have shown that AKT‐independent mechanisms of mTOR activation are involved in breast carcinogenesis (Tee *et al*., 2003)***.*** Recently, the mTOR inhibitors temsirolimus and everolimus and the PI3K inhibitors idelalisib and copanlisib have been approved by the FDA for clinical use in the treatment of a number of different cancers. Very recently, it was shown that early ctDNA dynamics in the commonly truncal mutations in *PIK3CA* may predict sensitivity to palbociclib, a CDK4/6 inhibitor (O'Leary *et al*., 2018a,2018b). Novel compounds with greater potency and selectivity, as well as improved therapeutic indices owing to reduced risks of toxicity, are clearly required. In addition, biomarkers that are predictive of a response, such as *PIK3CA* mutations for inhibitors of the PI3K catalytic subunit α isoform, must be identified and analytically and clinically validated (Janku *et al*., 2018).

*PIK3CA* mutational status has already been studied in CTCs (Bingham *et al*., 2017; Gasch *et al*., 2016; Markou *et al*., 2014; Schneck *et al*., 2013) and plasma‐ctDNA (Moynahan *et al*., 2017; Takeshita *et al*., 2017). When *PIK3CA* hotspot mutations were analyzed in CTCs isolated using a label‐free technology and corresponding ctDNA in 9 patients using 23 identical blood draws with colorectal cancer, mutational status of *PIK3CA* showed a match between CTCs and ctDNA in six blood draws and discordance in two, with 15 blood draws showing no mutations; however, in this study, PCR‐based Sanger sequencing (analytical sensitivity 7.5%) was used for mutation detection in CTCs and targeted panel NGS for cfDNA (Kidess‐Sigal *et al*., 2016). In another recent study, in a limited number of five metastatic breast cancer patients with more than 100 CTCs/7.5 mL PB (enumerated using the CellSearch), single EpCAM‐positive CTCs were isolated by DEPArray and compared with matched cfDNA and primary tumor tissue by targeted NGS for about 2,200 mutations in 50 cancer genes; according to this study, in these five patients, cfDNA profiles provided an accurate reflection of *PIK3CA* mutations seen in individual CTCs (Kidess‐Sigal *et al*., 2016; Shaw *et al*., 2017). According to these studies, the percentage of *PIK3CA* hotspot mutations in CTC and plasma‐ctDNA is around 30–40%. Our results indicate a much higher percentage of *PIK3CA*‐positive samples in both CTC and plasma‐ctDNA and this could possibly be explained by the higher analytical sensitivity of the molecular assay that we used (analytical sensitivity 0.05%) (Markou *et al*., 2014).

According to our results, in both early and metastatic BrCa, *PIK3CA* hotspot mutations were detected in the large majority of DNAs isolated from CellSearch^®^ cartridges but in a lower number of paired plasma‐ctDNA samples. It is interesting to note that in most cases where CTCs were detected by CellSearch^®^, *PIK3CA* hotspot mutations were identified either in ctDNA or in CTCs or both, in the early and metastatic BrCa setting. There were two cases where CTCs were detected at high numbers, but *PIK3CA* mutations were not detected in CTCs. Moreover, there were many cases where CTC were not detected by CellSearch^®^ but our downstream analysis in DNA material isolated from these cartridges was positive for *PIK3CA* mutations. This finding could be possibly explained by the fact that some EpCAM‐positive cells can be negative for CKs (8, 18, 19) due to EMT. These findings are in accordance with our previous results, where we identified *PIK3CA* hotspot mutations in EpCAM‐positive cells that were negative for *CK‐19* mRNA expression (Lampignano *et al*., 2017; Markou *et al*., 2014). We have also shown in one of our previous studies (Markou *et al*., 2018) by using exactly the same blood draws that we can detect a lot of molecular alterations in EpCAM‐positive CTCs, in identical samples that were ‘officially’ negative for CTCs when using the CellSearch.

We realize that we report a high percentage of *PIK3CA* mutations in primary tumors; however, in all cases where *PIK3CA* E545K hotspot mutation was detected in FFPEs, it was also detected in DNA isolated from CellSearch^®^ cartridges. However, we have to emphasize that the number of primary tumors that we tested was very low, since these are not randomly selected samples, but highly selected paired samples, where ctDNA and CTCs were also available, and our main aim was not to show the incidence of *PIK3CA* mutations in primary breast tumors but to compare the *PIK3CA* mutational status in primary tumors, CTCs and ctDNA. Our findings are in accordance with our previous paper (Markou *et al*., 2014), and we confirm again that there was no concordance in CTCs, ctDNA, and primary tumors.

Our aim was to evaluate the potential of our previously published assay for detecting *PIK3CA* hotspot mutations in DNAs isolated from CellSearch^®^ cartridges and paired plasma‐ctDNA samples in early and metastatic breast cancer patients. The patients’ selection was performed independently from the receptor status of these patients. Our aim was clearly not to provide evidence regarding the potential utility of *PIK3CA* mutation status by plasma‐ctDNA or CTC in predicting response to specific therapy. This is not a clinical study, and the samples analyzed were not selected based on the clinical characteristics of the patients. On the contrary, the BELLE‐2 and BELLE‐3 clinical trial studies included a large number of clinical samples, and patients’ selection was based on specific inclusion and exclusion criteria, demonstrating the incidence of *PIK3CA* mutations in specific target populations.

Our direct comparison study revealed for the first time that in early breast cancer, *PIK3CA* hotspot mutations are present in CTCs at a higher percentage before than after treatment, when compared to paired plasma‐ctDNA samples. This finding may suggest that in early BrCa, standard treatment was more often successful in targeting a number of cancer cells, thus leading to the release of DNA derived from these dying cells into the systemic circulation. However, in metastatic BrCa, the percentage of *PIK3CA* hotspot mutations in DNAs isolated from CellSearch^®^ cartridges was significantly higher in post‐treatment samples. This could be possibly explained by increased resistance to standard therapy which was not targeted therapy for *PIK3CA* hotspot mutations. Therefore, this nontargeted therapy seems to be ineffective to reduce the CTC/tumor burden in metastatic BrCa patients harboring *PIK3CA* hotspot mutations. In the same samples, the presence of *PIK3CA* hotspot mutations in plasma‐ctDNA was decreased after treatment, possibly indicating that the cells remained intact, hampering the release of ctDNA in peripheral blood. The concordance of *PIK3CA* mutations between CTCs and ctDNA was relatively poor in early breast cancer (48.2%) and higher in the metastatic setting (66.6%).

Since the high incidence of mutations found could raise questions, we performed a comparison study in the same samples using a commercially available highly sensitive methodology for *PIK3CA* mutations based on ddPCR, for ctDNA samples and CTCs that were available. Our results revealed a high concordance between our assay and ddPCR (81.1%, *P* = 0.001).

Our results in CTCs could only be compared with similar studies using highly sensitive methodologies, like ddPCR; however, till now so highly sensitive methodologies have not been applied in CTCs isolated using the CellSearch system. In the study of Schneck *et al*., the mutation rate reported for *PIK3CA* mutations in CTCs was only 15.8%. This percentage is much lower than the one that we report; however, this discrepancy can be clearly explained by the much lower analytical sensitivity of the SNaPshot methodology that is 5 to 10% (Hurst *et al*., 2009). Our results in ctDNA of metastatic breast cancer patients are in agreement with a very recent study by O'Leary *et al*. (2018b) where a percentage of 56.3% (9/16) is reported for *PIK3CA* mutations, in ctDNA samples, since we also report for metastatic breast cancer a similar percentage, even lower, more specifically 47.9% (35/73) in ctDNA samples. In the independent patient group, *PIK3CA* hotspot mutations were present at high frequencies (59.5%) in DNA samples isolated from CellSearch^®^ cartridges. In most cases where CellSearch^®^ detected at least one CTC, *PIK3CA* hotspot mutations were also identified. However, in 3 cases *PIK3CA* hotspot mutations were also detected in samples that were found negative for CTCs according to the CellSearch^®^. These data are in concordance with our previous results, indicating that *PIK3CA* mutations are also present in EpCAM‐positive cells where CellSearch gave negative results.

## 5 Conclusions

We performed for the first time a direct comparison study on the presence of *PIK3CA* hotspot mutations in CTCs and plasma‐ctDNA using identical blood draws and a highly sensitive and analytically validated methodology. Our results indicate that: (a) *PIK3CA* hotspot mutations are present at high frequencies in DNAs isolated from CellSearch^®^ cartridges and paired plasma‐ctDNA both in early and metastatic BrCa, (b) the concordance between plasma‐ctDNA and CTCs is higher in the metastatic setting, (c) *PIK3CA* mutational status significantly changes after therapeutic intervention, and (d) *PIK3CA* mutation detection in CTCs and plasma‐ctDNA provides complementary information. Our data reflect significant differences in CTCs and ctDNA before and after treatment and point toward the necessity of following patients over time using an integrated liquid biopsy approach.

## Conflict of interest

The authors declare no conflict of interest.

## Author Contributions

ET and AM performed the experiments. EP performed the experiments in the CellSearch. ET, AM, and EL wrote the manuscript. ET, AM, AK, AP, DM, VG, and EL edited the manuscript. AK, AP, DM, and VG collected the clinical specimens. EL designed the study and supervised the experimental procedures.

## Supporting information


**Fig S1.** Independent group: *PIK3CA* hotspot mutations in DNA isolated from CellSearch^®^ cartridges, before and after treatment from metastatic BrCa patients (*n* = 17).

**Table S1.** Clinicopathological characteristics of early and metastatic BrCa patients used in the direct comparison study.
**Table S2.** Correlation of *PIK3CA* hotspot mutations and clinicopathological characteristics of early and metastatic BrCa patients used in the direct comparison study.
