# Supplementary material for: PIK3CA hotspot mutations in circulating tumor cells and paired circulating tumor DNA in breast cancer: a direct comparison study
Source: Mol Oncol. 2019 Sep 30;13(12):2515–30. doi: 10.1002/1878-0261.12540 (PMC6887588; doi:10.1002/1878-0261.12540)
Supplement: Supplementary file 1 — Fig S1. Independent group: PIK3CA hotspot mutations in DNA isolated from CellSearch® cartridges, before and after treatment from metastatic BrCa patients (n = 17). [file MOL2-13-2515-s001.pptx]

## Slide 1
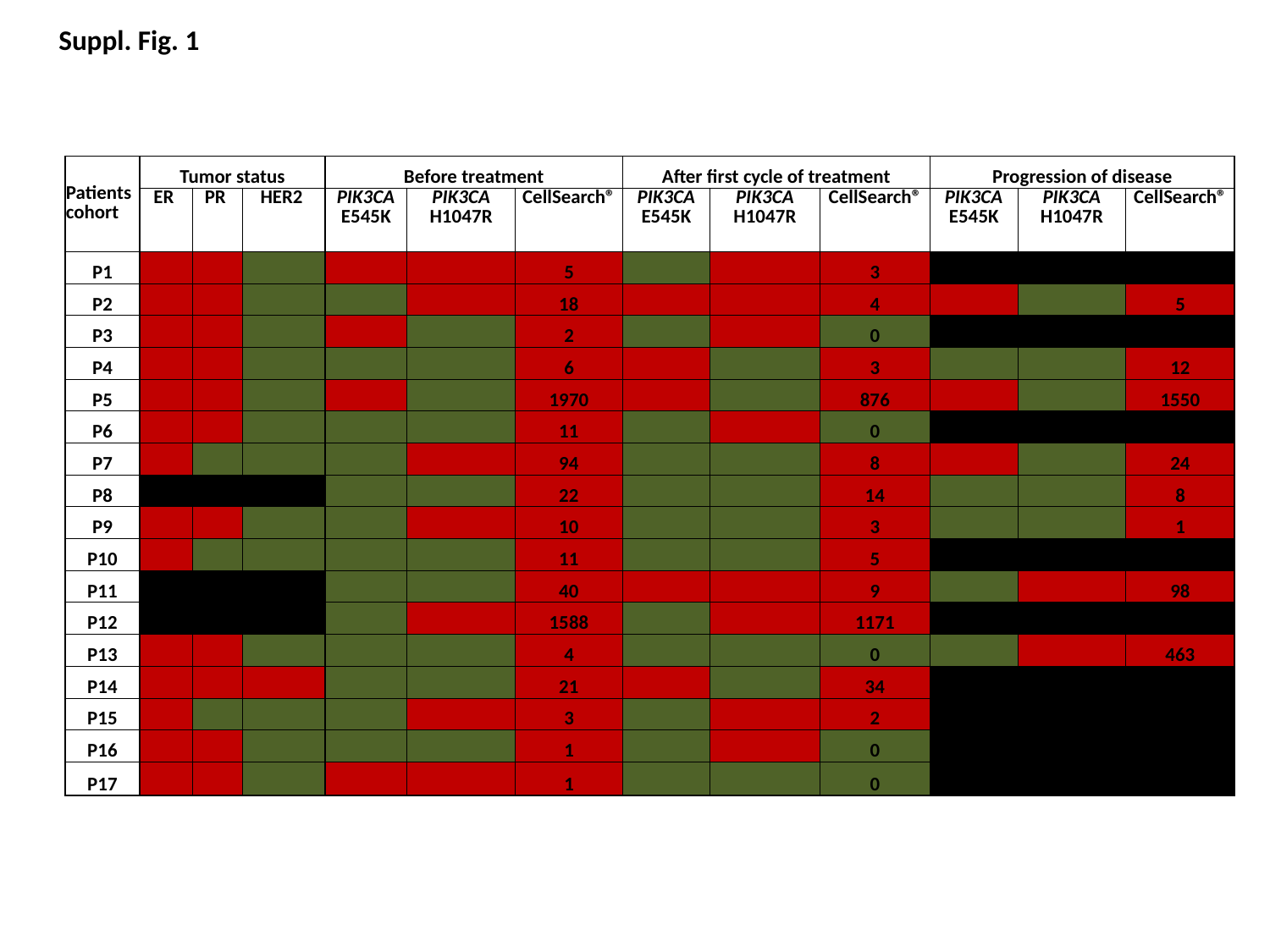

Suppl. Fig. 1
| Patients cohort | Tumor status | | | Before treatment | | | After first cycle of treatment | | | Progression of disease | | |
| --- | --- | --- | --- | --- | --- | --- | --- | --- | --- | --- | --- | --- |
| | ER | PR | HER2 | PIK3CA E545K | PIK3CA H1047R | CellSearch® | PIK3CA E545K | PIK3CA H1047R | CellSearch® | PIK3CA E545K | PIK3CA H1047R | CellSearch® |
| P1 | | | | | | 5 | | | 3 | | | |
| P2 | | | | | | 18 | | | 4 | | | 5 |
| P3 | | | | | | 2 | | | 0 | | | |
| P4 | | | | | | 6 | | | 3 | | | 12 |
| P5 | | | | | | 1970 | | | 876 | | | 1550 |
| P6 | | | | | | 11 | | | 0 | | | |
| P7 | | | | | | 94 | | | 8 | | | 24 |
| P8 | | | | | | 22 | | | 14 | | | 8 |
| P9 | | | | | | 10 | | | 3 | | | 1 |
| P10 | | | | | | 11 | | | 5 | | | |
| P11 | | | | | | 40 | | | 9 | | | 98 |
| P12 | | | | | | 1588 | | | 1171 | | | |
| P13 | | | | | | 4 | | | 0 | | | 463 |
| P14 | | | | | | 21 | | | 34 | | | |
| P15 | | | | | | 3 | | | 2 | | | |
| P16 | | | | | | 1 | | | 0 | | | |
| P17 | | | | | | 1 | | | 0 | | | |
