# Supplementary material for: PIK3CA hotspot mutations in circulating tumor cells and paired circulating tumor DNA in breast cancer: a direct comparison study
Source: Mol Oncol. 2019 Sep 30;13(12):2515–30. doi: 10.1002/1878-0261.12540 (PMC6887588; doi:10.1002/1878-0261.12540)
Supplement: Supplementary file 2 — Table S1. Clinicopathological characteristics of early and metastatic BrCa patients used in the direct comparison study. Table S2. Correlation of PIK3CA hotspot mutations and clinicopathological characteristics of early and metastatic BrCa patients used in the direct comparison study. [file MOL2-13-2515-s002.docx]

**Supplementary Table 1:** Clinicopathological characteristics of early and metastatic BrCa patients used in the direct comparison study

| **Characteristics** | **Early BrCa (n=43)** | **Metastatic BrCa (n=16)** |
| --- | --- | --- |
| **Age (years)** |  |  |
| ˂50 | 16 | 1 |
| ≥50 | 26 | 14 |
| Unknown | 1 | 1 |
| **Menopausal status** |  |  |
| pre | 15 | 2 |
| post | 10 | 12 |
| Unknown | 8 | 2 |
| **Stage** |  |  |
| I and II | 22 | 6 |
| III | 19 | 8 |
| Unknown | 2 | 2 |
| **Tumor size** |  |  |
| ≤2 cm | 19 | 1 |
| 2–5 cm | 20 | 4 |
| >5 cm | 2 | 3 |
| Unknown | 2 | 8 |
| **Molecular subtype** |  |  |
| Luminal | 25 | 6 |
| HER2 pos | 8 | 6 |
| Triple negative | 10 | 3 |
| Unknown | 0 | 1 |
| **ER status** |  |  |
| pos | 28 | 10 |
| neg | 14 | 5 |
| unknown | 1 | 1 |
| **PR status** |  |  |
| pos | 23 | 8 |
| neg | 17 | 6 |
| unknown | 3 | 2 |
| **HER2 status** |  |  |
| pos | 8 | 6 |
| neg | 35 | 9 |
| unknown | 0 | 1 |
| **Ki 67** |  |  |
| ˂20% | 15 | 4 |
| ≥20% | 22 | 9 |
| unknown | 6 | 3 |

**Supplementary Table 2:** Correlation of *PIK3CA* hotspot mutations and clinicopathological characteristics of early and metastatic BrCa patients used in the direct comparison study

| **Patient Characteristics** | **Metastatic BrCa (n=16)** | **Plasma-ctDNA** | **CTC** | **ctDNA and/or CTC** |
| --- | --- | --- | --- | --- |
| **Age (years)** |  |  |  |  |
| ˂55 | 4 | 3 | 4 | 4 |
| ≥55 | 11 | 9 | 7 | 10 |
| Unknown | 1 | 0 | 0 | 0 |
|  |  | P=0.637 | P=0.330 | P=0.733 |
| **GRADE** |  |  |  |  |
| I and II | 6 | 6 | 6 | 7 |
| III | 8 | 6 | 5 | 7 |
| Unknown | 2 | 0 | 0 | 0 |
|  |  | P=0.554 | P=0.154 | P=0.533 |
| **Tumor size** |  |  |  |  |
| ≤2 cm | 2 | 1 | 2 | 2 |
| 2–5 cm | 2 | 2 | 2 | 2 |
| >5 cm | 3 | 3 | 2 | 3 |
|  |  | P=0.108 | P=0.641 | P=0.386 |
| **ER status** |  |  |  |  |
| pos | 10 | 8 | 8 | 9 |
| neg | 5 | 4 | 3 | 5 |
| unknown | 1 | 0 | 0 | 0 |
|  |  | P=0.758 | P=0.275 | P=0.667 |
| **PR status** |  |  |  |  |
| pos | 8 | 7 | 7 | 7 |
| neg | 6 | 5 | 4 | 7 |
| unknown | 2 | 0 | 0 | 0 |
|  |  | P=0.446 | P=0.385 | P=0.533 |
| **HER2 status** |  |  |  |  |
| pos | 6 | 5 | 4 | 6 |
| neg | 9 | 7 | 7 | 8 |
|  | 1 | 0 | 0 | 0 |
|  |  | P=0.659 | P=0.725 | P=0.600 |
| **Ki 67** |  |  |  |  |
| ˂14% | 5 | 5 | 4 | 5 |
| ≥14% | 10 | 7 | 7 | 9 |
| unknown | 2 | 0 | 0 | 0 |
|  |  | P=0.264 | P=0.330 | P=0.667 |
| **Patient Characteristics** | **Early BrCa (n=43)** | **Plasma-ctDNA** | **CTC** | **ctDNA and/or CTC** |
| **Age (years)** |  |  |  |  |
| ˂55 | 21 | 6 | 16 | 19 |
| ≥55 | 21 | 9 | 14 | 16 |
| Unknown | 1 | 0 | 0 | 0 |
|  |  | P=0.299 | P=0.287 | P=0.135 |
| **GRADE** |  |  |  |  |
| I and II | 23 | 6 | 15 | 18 |
| III | 19 | 9 | 14 | 16 |
| Unknown | 1 | 0 | 0 | 0 |
|  |  | P=0.134 | P=0.401 | P=0.466 |
| **Tumor size** |  |  |  |  |
| ≤2 cm | 20 | 10 | 14 | 17 |
| 2–5 cm | 20 | 5 | 14 | 16 |
| >5 cm | 2 | 0 | 1 | 1 |
|  |  | P=0.143 | P=0.837 | P=0.480 |
| **ER status** |  |  |  |  |
| pos | 28 | 10 | 19 | 23 |
| neg | 14 | 5 | 10 | 11 |
| unknown | 1 | 0 | 0 | 0 |
|  |  | P=0.629 | P=0.553 | P=0.543 |
| **PR status** |  |  |  |  |
| pos | 24 | 8 | 18 | 20 |
| neg | 18 | 7 | 11 | 14 |
| unknown | 1 | 0 | 0 | 0 |
|  |  | P=0.480 | P=0.265 | P=0.473 |
| **HER2 status** |  |  |  |  |
| pos | 8 | 4 | 5 | 7 |
| neg | 35 | 11 | 25 | 28 |
|  |  | P=0.275 | P=0.458 | P=0.533 |
| **Ki 67** |  |  |  |  |
| ˂14% | 13 | 4 | 8 | 9 |
| ≥14% | 29 | 11 | 21 | 25 |
| unknown | 1 | 0 | 0 | 0 |
|  |  | P=0.466 | P=0.360 | P=0.190 |
